# Supplementary material for: A Critical View of the Application of the APEX Software (Aqueous Photochemistry of Environmentally-Occurring Xenobiotics) to Predict Photoreaction Kinetics in Surface Freshwaters
Source: Molecules. 2019 Dec 18;25(1):9. doi: 10.3390/molecules25010009 (PMC7017383; doi:10.3390/molecules25010009)
Supplement: Supplementary file 1 [file molecules-25-00009-s001.zip › 3.2.4_gcc-4.4.0.zip!/3.2.4_gcc-4.4.0/3.2.4_gcc-4.4.0/doc/octave/html/interpreter/A-Sample-Command-Description.html]

A Sample Command Description - Untitled


Next: A Sample Variable Description,
Previous: A Sample Function Description,
Up: Format of Descriptions


---

##### 1.3.5.2 A Sample Command Description

Command descriptions have a format similar to function descriptions,
except that the word `Function' is replaced by `Command'. Commands are
functions that may be called without surrounding their arguments in
parentheses. For example, here is the description for Octave's
`cd` command:

— Command: **cd** dir  
— Command: **chdir** dir  
> Change the current working directory to dir. For example,
> `cd ~/octave` changes the current working directory to
> ~/octave. If the directory does not exist, an error message is
> printed and the working directory is not changed.
